# Author Correction: The rapid electrochemical activation of MoTe_2_ for the hydrogen evolution reaction

**DOI:** 10.1038/s41467-019-13337-5

**Published:** 2019-11-20

**Authors:** Jessica C. McGlynn, Torben Dankwort, Lorenz Kienle, Nuno A. G. Bandeira, James P. Fraser, Emma K. Gibson, Irene Cascallana-Matías, Katalin Kamarás, Mark D. Symes, Haralampos N. Miras, Alexey Y. Ganin

**Affiliations:** 1 School of Chemistry, University of Glasgow, Glasgow, G12 8QQ UK; 2 Institute for Materials Science, University of Kiel, Kaiserstraße 2, 24143 Kiel, Germany; 3 BioISI–BioSystems and Integrative Sciences Institute and Centro de Química e Bioquímica, C8-Faculdade de Ciências da Universidade de Lisboa, Campo Grande, 1749-016 Lisboa, Portugal; 4 Centro de Química Estrutural, Instituto Superior Técnico, Universidade de Lisboa, Av. Rovisco Pais 1, 1049-001 Lisboa, Portugal; 5 Institute for Solid State Physics and Optics, Wigner Research Centre for Physics, Hungarian Academy of Sciences, P.O. Box 49, Budapest, 1525 Hungary

**Keywords:** Electrocatalysis, Electrocatalysis, Two-dimensional materials

Correction to: *Nature Communications* 10.1038/s41467-019-12831-0, published online 29 October 2019.

The original version of this Article contained an error in the spelling of the author Haralampos N. Miras, which was incorrectly given as Harry N. Miras. This has now been corrected in both the PDF and HTML versions of the Article.

