# Supplementary material for: The rapid electrochemical activation of MoTe2 for the hydrogen evolution reaction
Source: Nat Commun. 2019 Oct 29;10:4916. doi: 10.1038/s41467-019-12831-0 (PMC6820771; doi:10.1038/s41467-019-12831-0)
Supplement: Supplementary file 1 — Supplementary Info [file 41467_2019_12831_MOESM1_ESM.pdf]

Supplementary Information

The rapid electrochemical activation of $MoTe_2$ for the hydrogen evolution reaction

McGlynn *et al*.

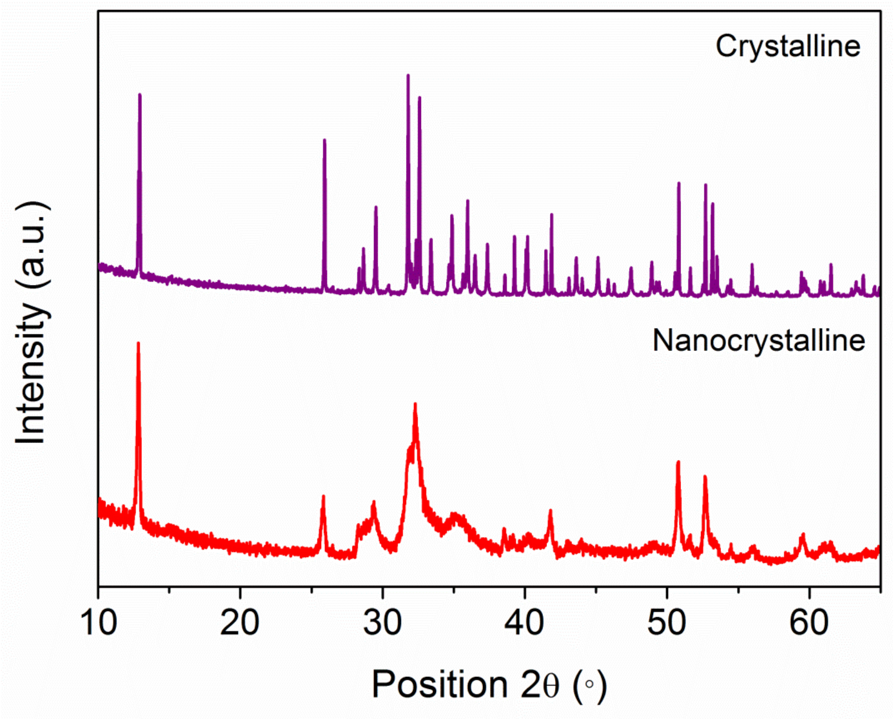

Supplementary Figure 1: Powder X-Ray Diffraction patterns of crystalline 1T'-$MoTe_2$ (top) and nanocrystalline 1T'-$MoTe_2$ (bottom).

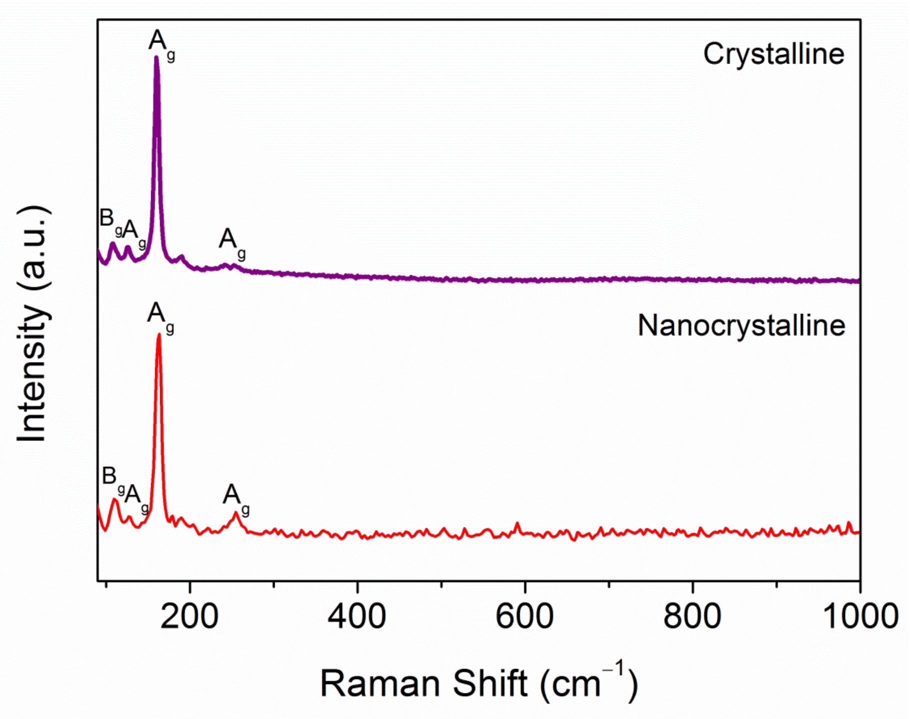

Supplementary Figure 2: Wide-range Raman spectra with the four main vibrational modes highlighted of crystalline and nanocrystalline 1T'-$MoTe_2$.

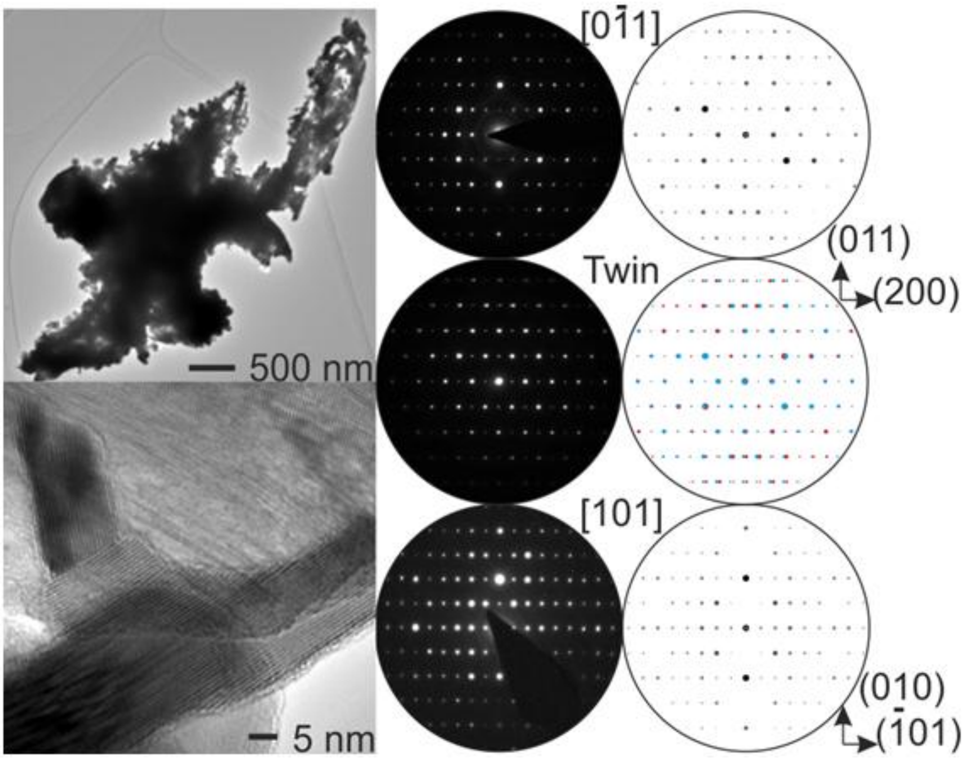

Supplementary Figure 3: TEM studies of nanocrystalline 1T'-$MoTe_2$. Representative low **a** and high **b** magnification TEM images of the nanocrystalline 1T'-$MoTe_2$. The corresponding electron diffraction patterns along different directions crystal oprientations are shown in **c-e**.

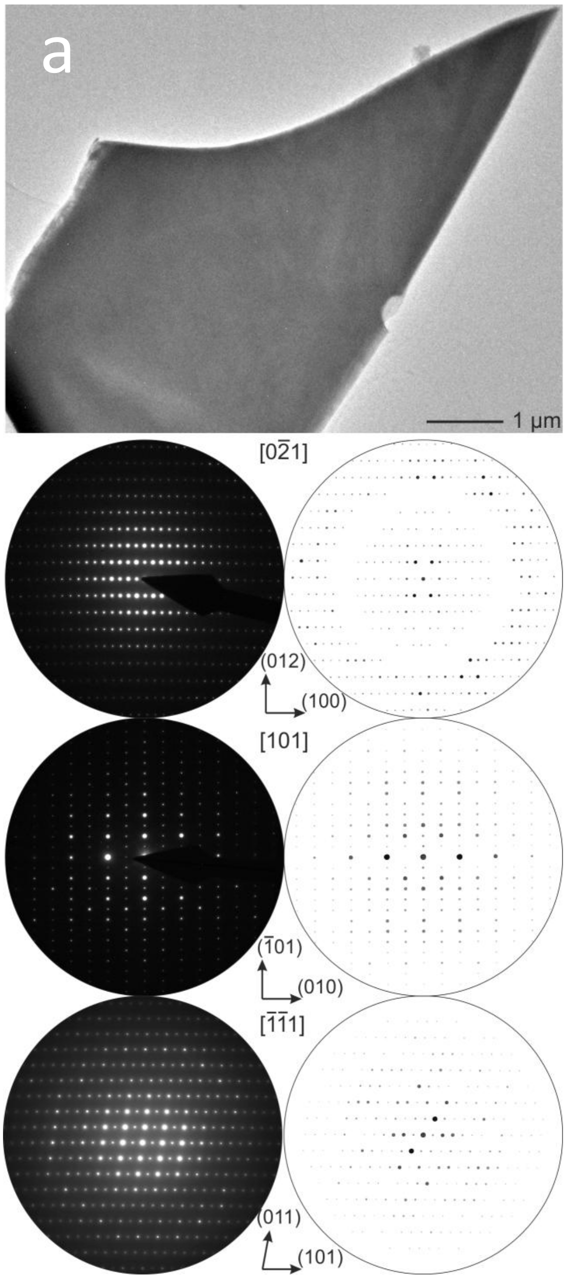

Supplementary Figure 4: TEM studies of crystalline 1T'-$MoTe_2$. Representative low magnification TEM image of crystalline 1T'-$MoTe_2$ **a** and the corresponding electron diffraction patterns for different crystal orientations are shown in **b-d**.

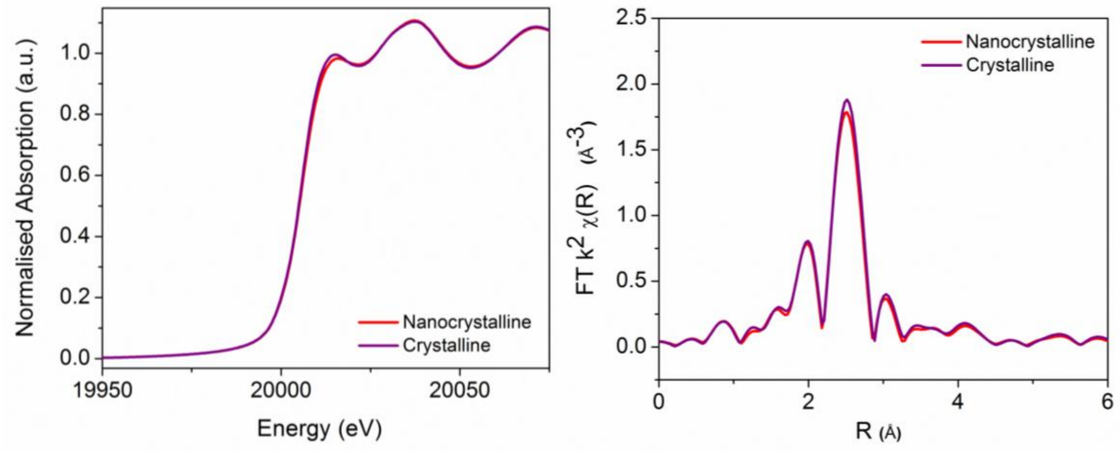

Supplementary Figure 5: Comparison of EXAFS data collected for nanocrystalline and crystalline 1T'-$MoTe_2$. **right** Normalised XANES spectra and **left** the k^2-weighted Fourier Transform of crystalline 1T'-$MoTe_2$ (purple) and nanocrystalline 1T'-$MoTe_2$ (red).

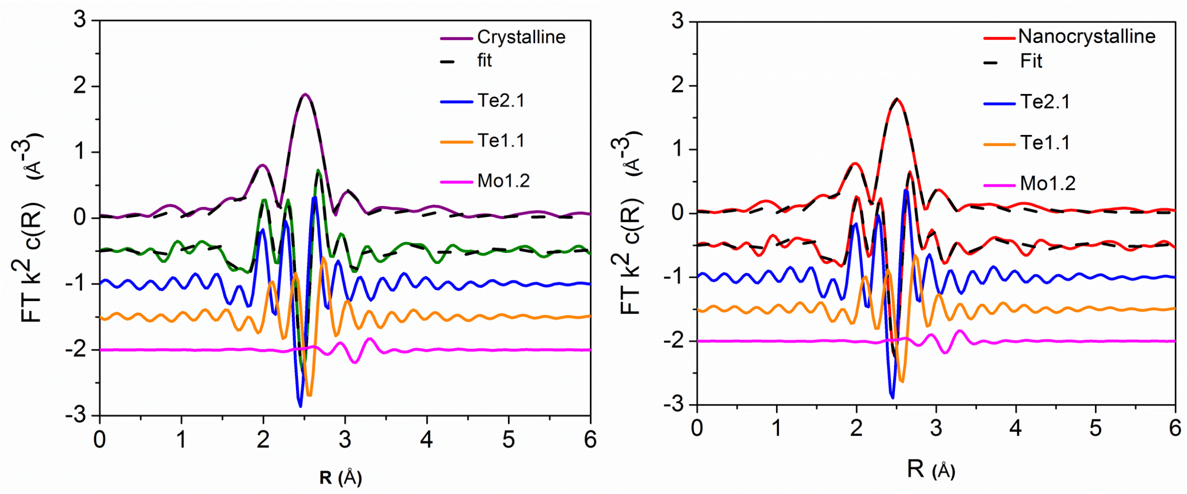

Supplementary Figure 6: Fourier transform data for crystalline and nanocrystalline 1T'-$MoTe_2$. **left** The magnitude and imaginary components of the non-phase corrected k^2 weighted Fourier transform data and fits of the crystalline 1T'-$MoTe_2$ and the imaginary components of each scattering path used. **right** The magnitude and imaginary components of the non-phase corrected k^2 weighted Fourier transform data and fits of the nanocrystalline 1T'-$MoTe_2$ and the imaginary components of each scattering path used.

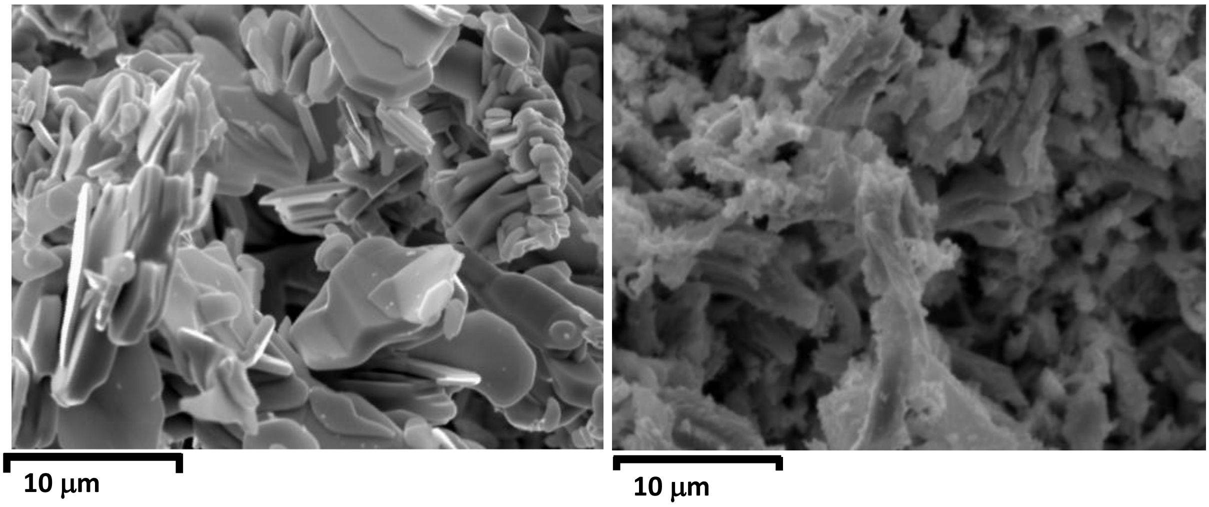

Supplementary Figure 7: Morphololology of nanocrystalline and crystalline 1T'-$MoTe_2$. **left** SEM images of the crystalline 1T'-$MoTe_2$ and **right** nanocrystalline 1T'-$MoTe_2$ on the micrometre scale, clearly showing that these are bulk materials.

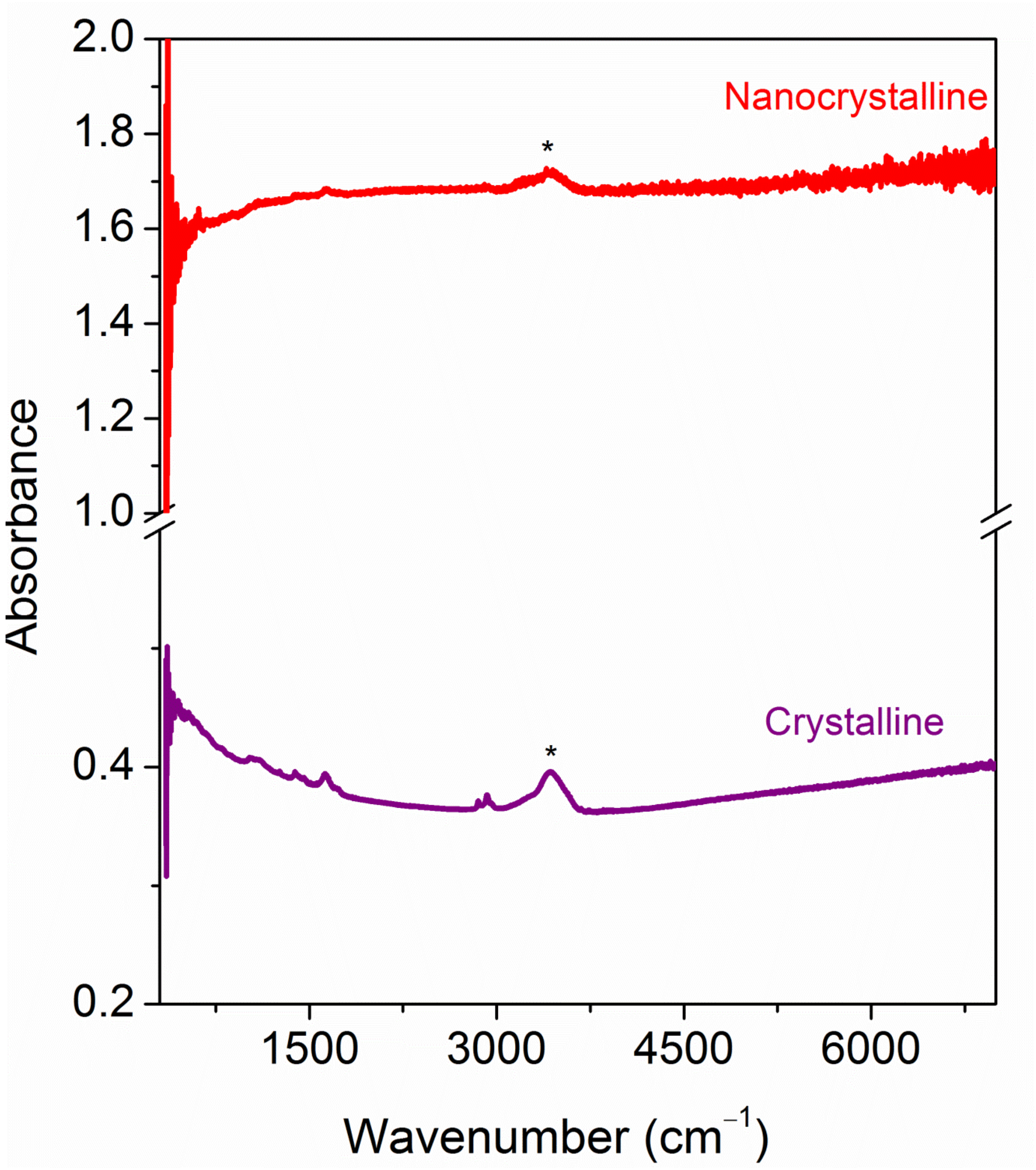

Supplementary Figure 8: Infrared spectra of the nanocrystalline and crystalline 1T'-$MoTe_2$ phases, respectively. The features denoted by asterisks indicate atmospheric water peaks.

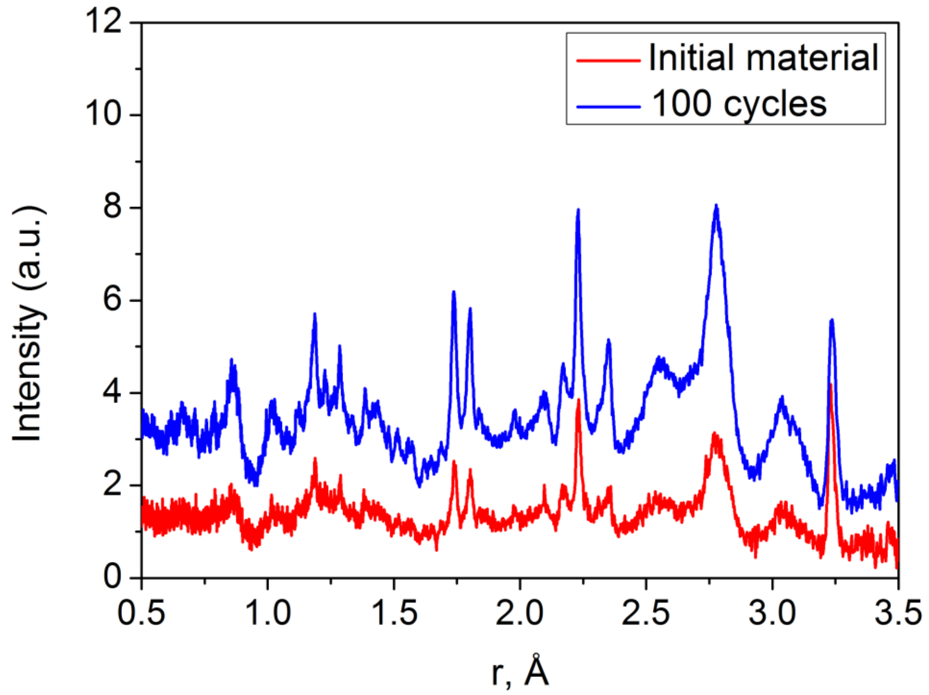

Supplementary Figure 9: Neutron scattering data from the $MoTe_2$ sample before and after activation in 1M D_2SO_4 in D_2O. The similarity of the profiles excludes the possible intercalation of protons between the layers of $MoTe_2$ as the result of the activation process.

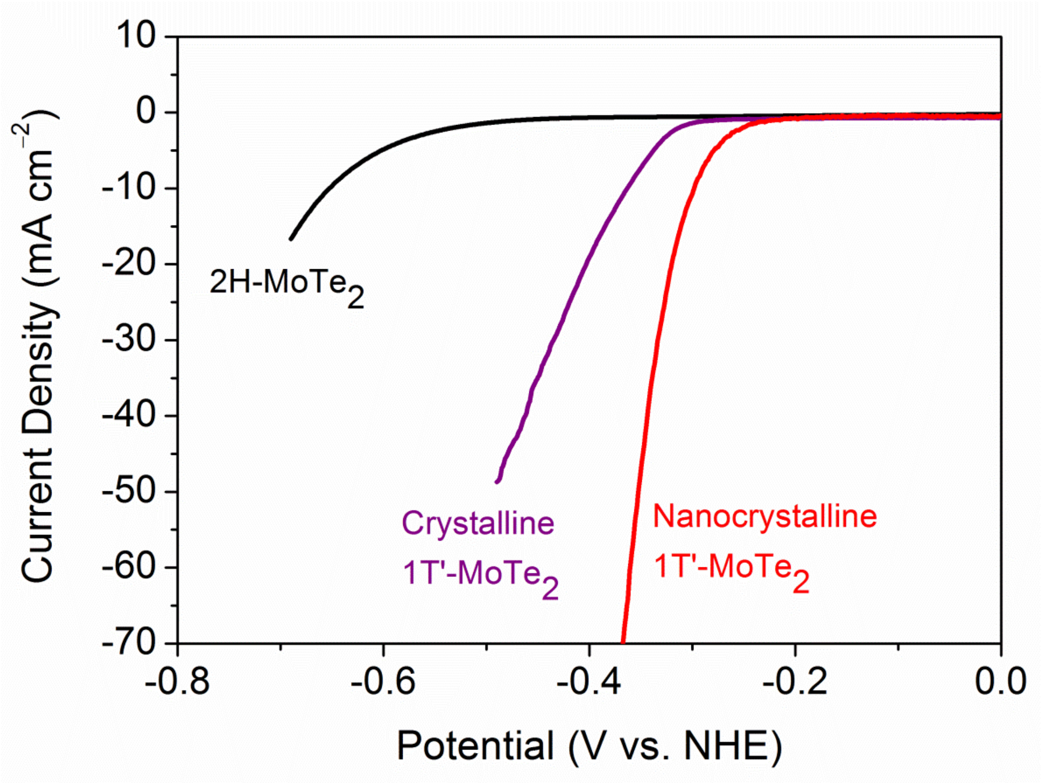

Supplementary Figure 10: Comparison of the current densities achieved by 2H-MoTe$_2$, crystalline 1T'-MoTe$_2$ and nanocrystalline 1T'-MoTe$_2$ catalysts in 1 M H_2SO_4. Catalysts were prepared on a glassy carbon working electrode as described in the experimental section. Carbon felt and 3 M Ag/AgCl were used as the counter and reference electrodes, respectively.

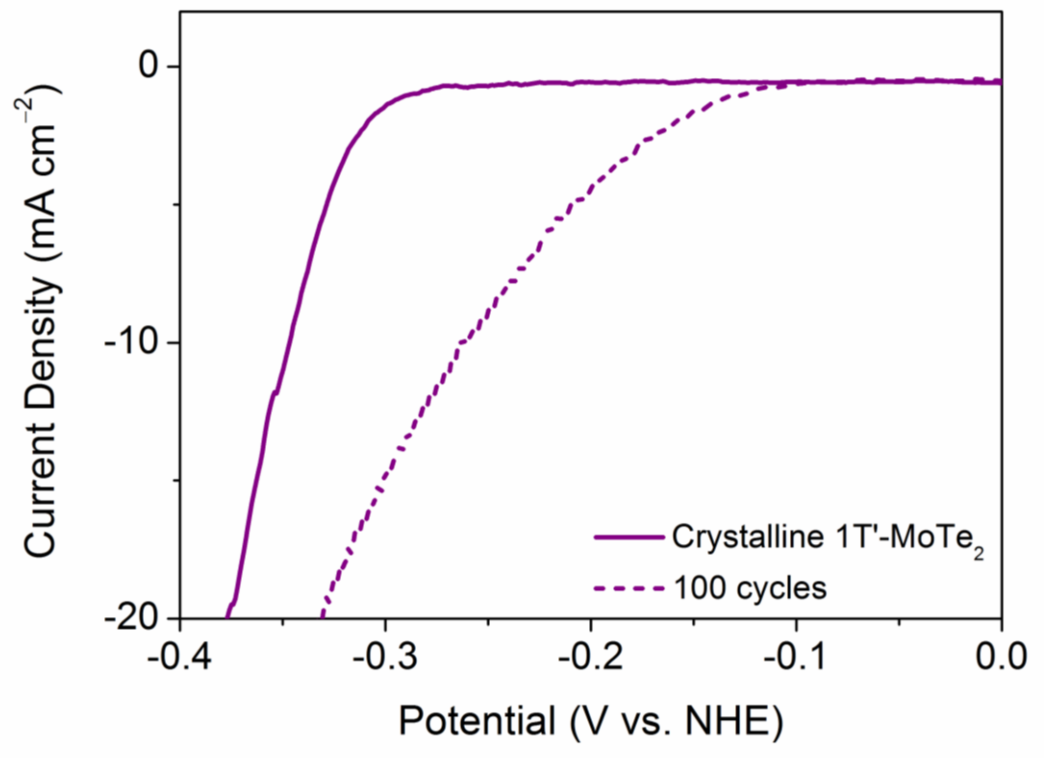

Supplementary Figure 11: Comparison of the current densities achieved by the crystalline 1T'-$MoTe_2$ before and after 100 cycles in 1 M H_2SO_4. An activation is observed which results in an improvement of overpotential at j = 10 mA cm^{-2} from 347 mV to 261 mV. Catalysts were prepared on a glassy carbon working electrode as described in the experimental section. Carbon felt and 3 M Ag/AgCl were used as the counter and reference electrodes, respectively.

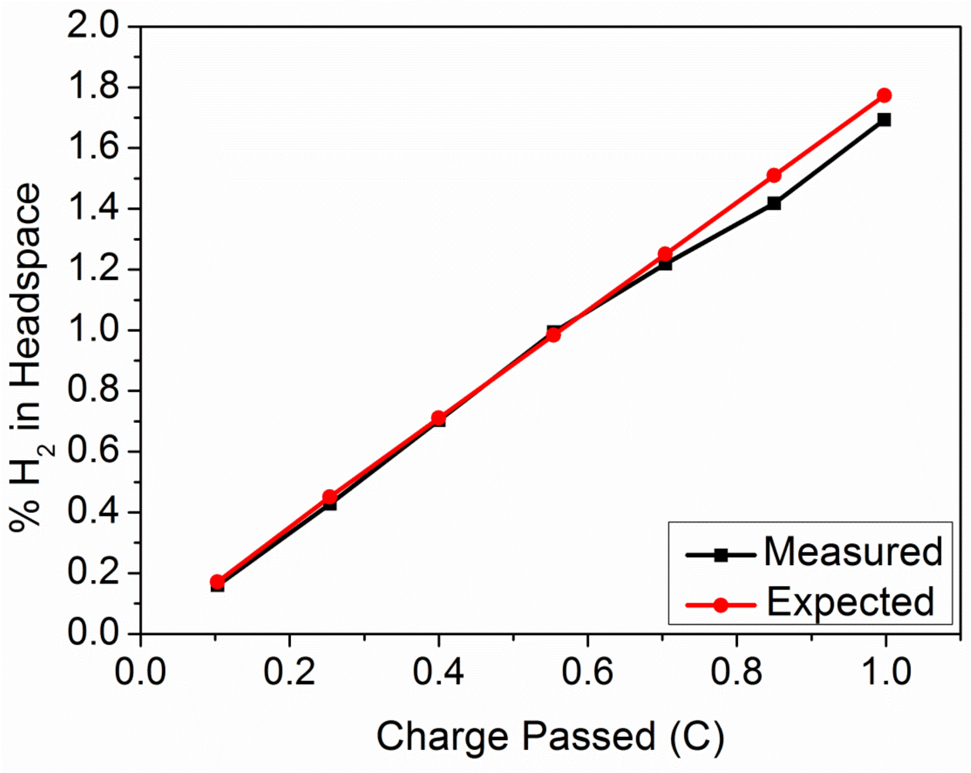

Supplementary Figure 12: A representative trace showing gas chromatographic analysis of the single cell headspace during electrolysis of nanocrystalline 1T'-$MoTe_2$.

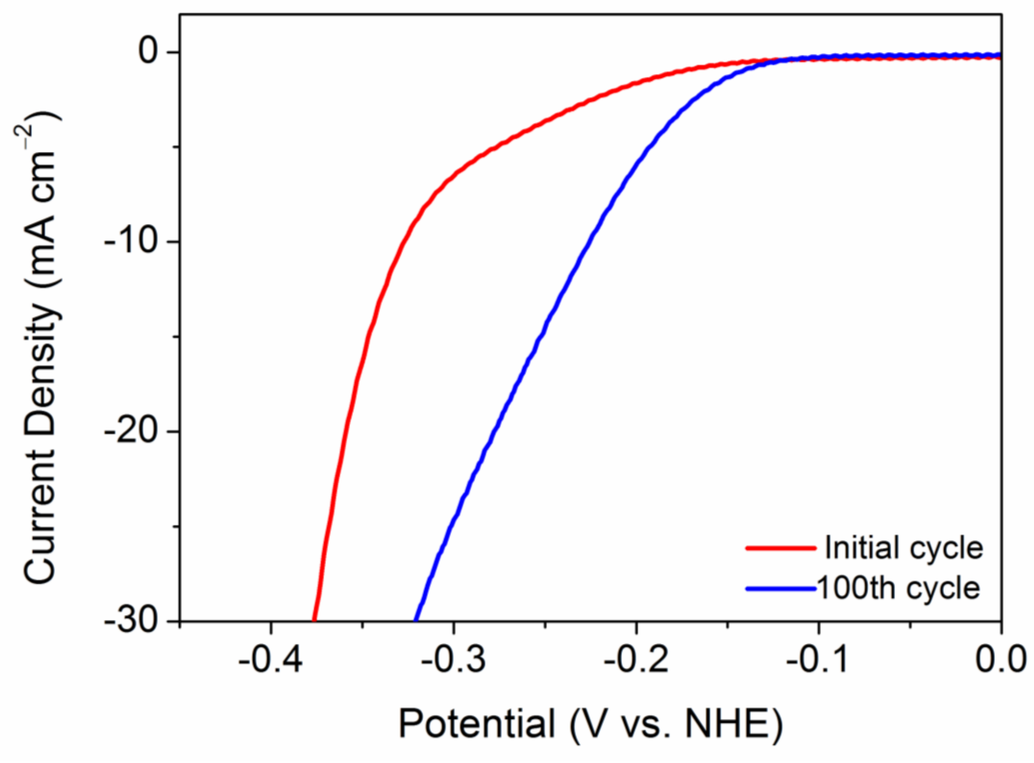

Supplementary Figure 13: Comparison of the current densities achieved by nanocrystalline 1T'-$MoTe_2$ before and after 100 cycles in 1 M H_2SO_4 using saturated Hg/Hg_2SO_4 as the reference electrode. The use of saturated Hg/Hg_2SO_4 as the reference electrode rules out leakage of silver from the Ag/AgCl reference electrode as the source of improved performance.

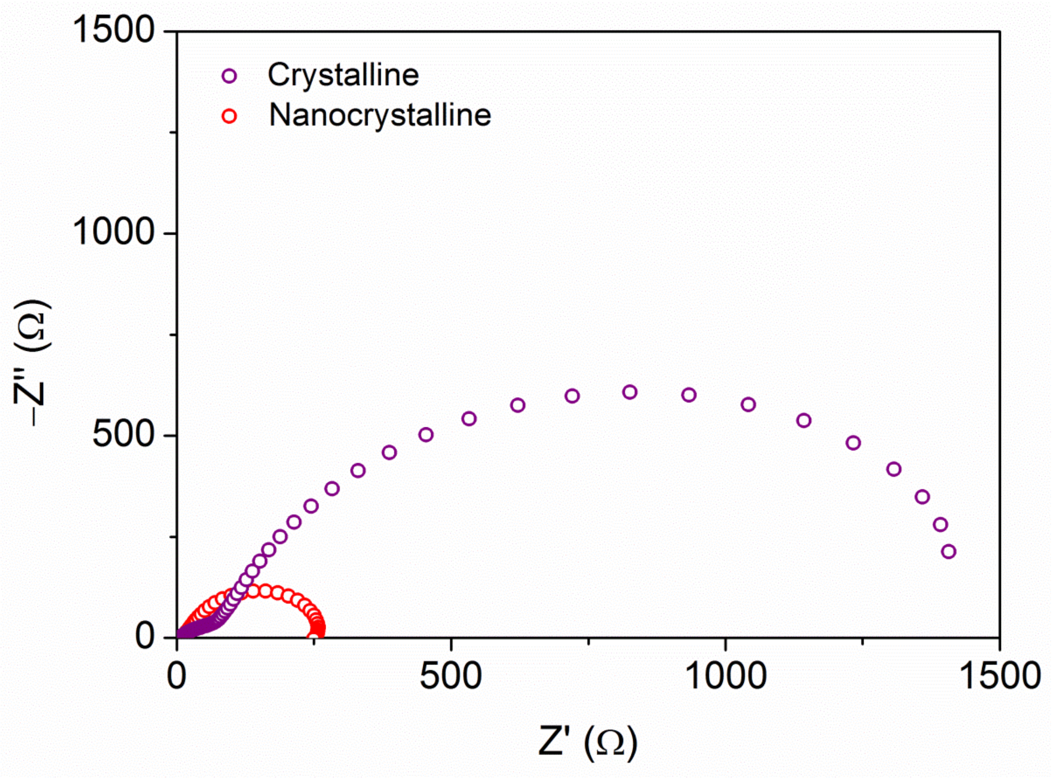

Supplementary Figure 14: Nyquist plots showing electrochemical impedance spectroscopy on the nanocrystalline and crystalline 1T'-$MoTe_2$ at –300 mV (vs. NHE). Uncompensated resistances were calculated as 8.9 and 8.1 Ω for the crystalline and nanocrystalline materials, respectively. This corresponds well with the iR compensation function on the potentiostat which gave values of 9.0 and 9.8 Ω.

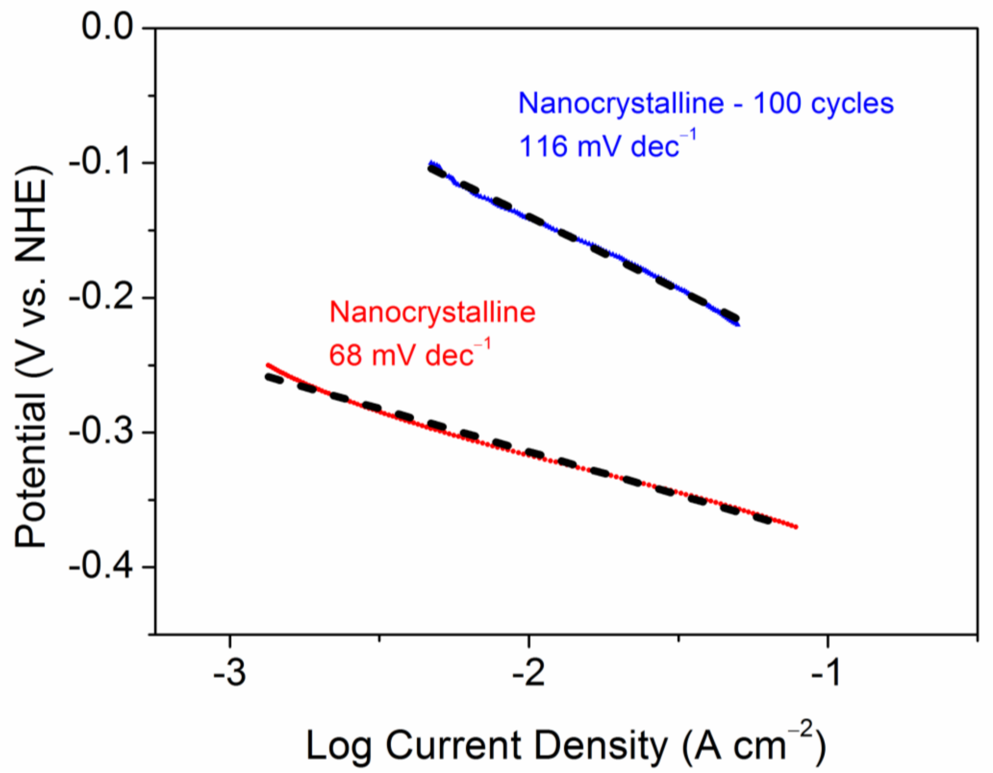

Supplementary Figure 15: Tafel plots and corresponding Tafel slopes of nanocrystalline 1T'-$MoTe_2$ before and after 100 cycles. Dashed lines are provided as a guide to the eye. All current densities have been corrected for resistance.

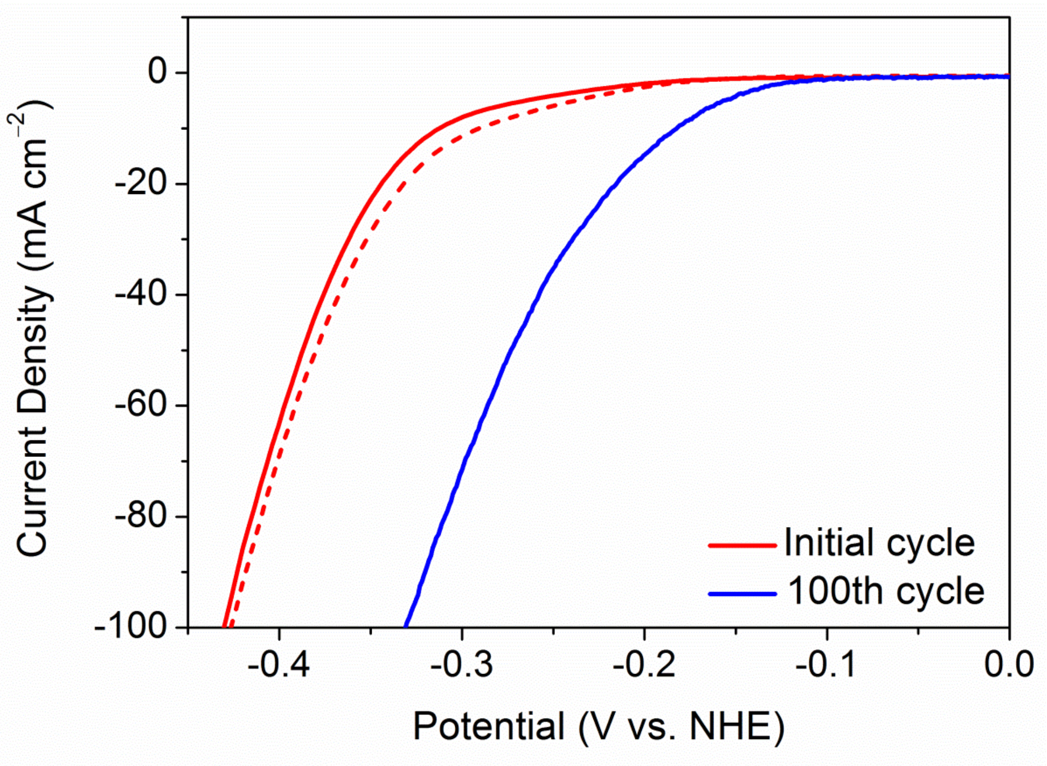

Supplementary Figure 16: Comparison of the current densities achieved by nanocrystalline 1T'-$MoTe_2$ before and after 100 cycles in 1 M H_2SO_4. The red dashed line illustrates the current densities achieved immediately after the 100 cycle scan, and shows the overpotential to return to its original value as the application of continuous cycling under reducing potentials is stopped.

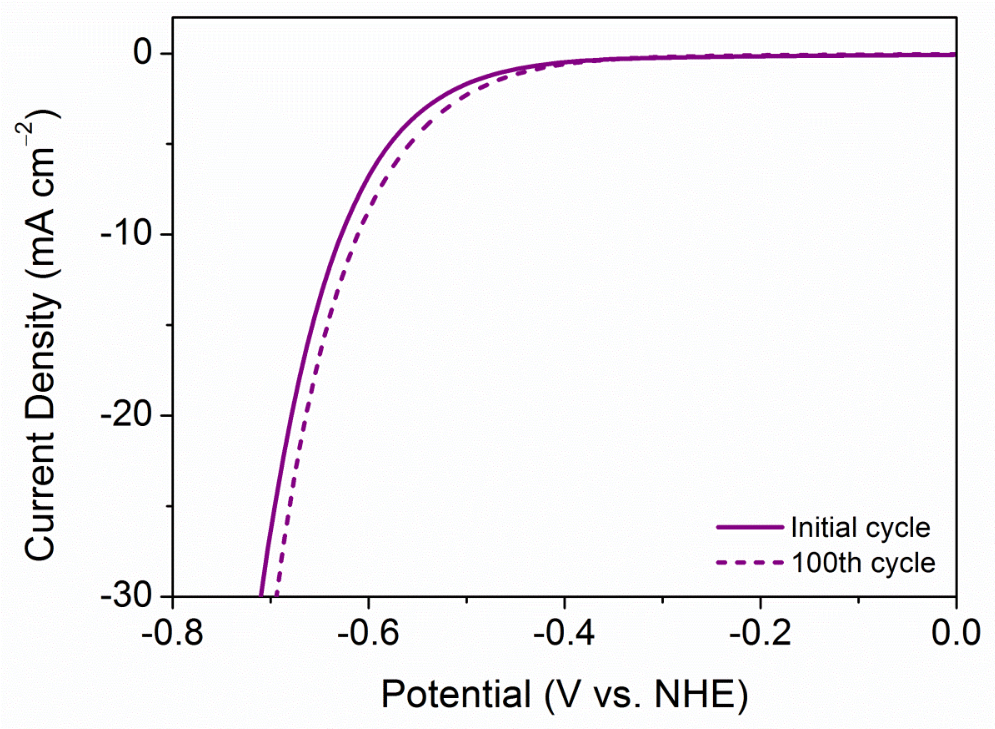

Supplementary Figure 17: Comparison of the current densities achieved by semiconducting 2H-$MoTe_2$ before and after 100 cycles in 1 M H_2SO_4.

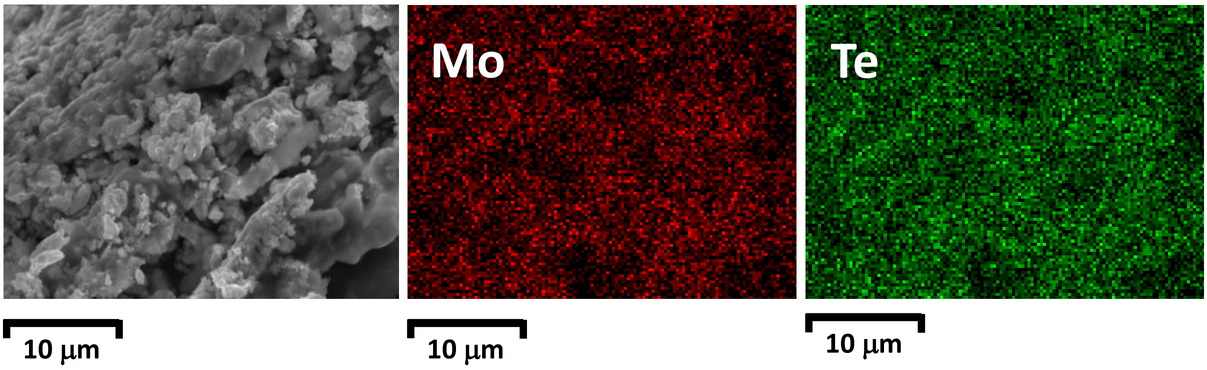

Supplementary Figure 18: SEM and EDX mapping images of nanocrystalline 1T'-$MoTe_2$ after 100 cycles. **left** SEM images of the nanocrystalline 1T'-$MoTe_2$ taken after 100 cycles ; **middle** EDX mapping image of Mo and **right** Te showing their equal distribution throughout the material.

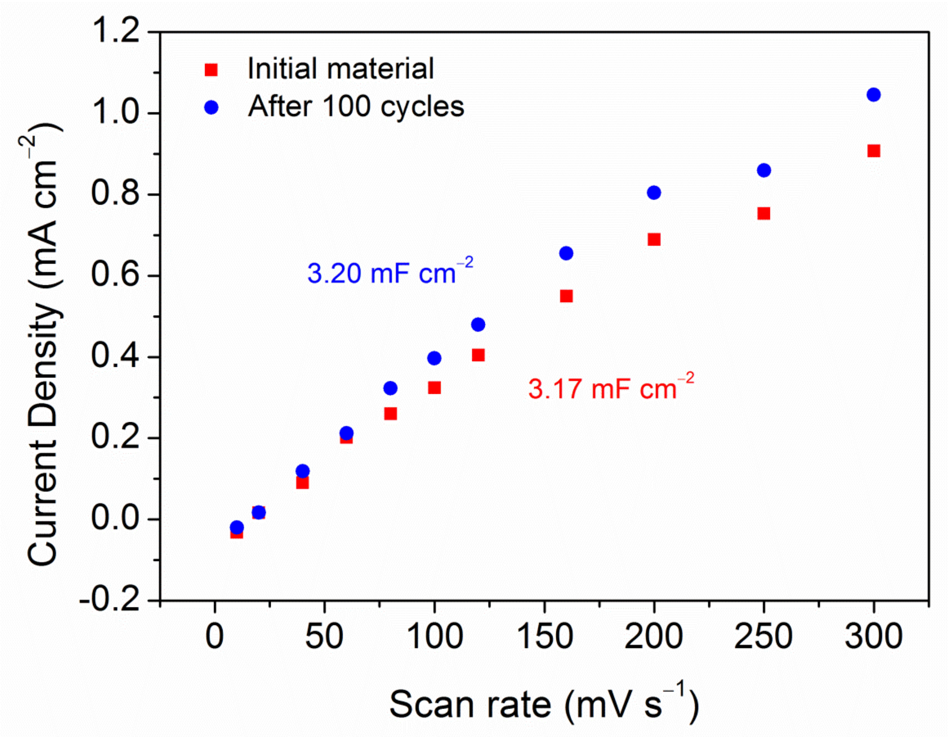

Supplementary Figure 19: Current density differences of nanocrystalline 1T'-$MoTe_2$ before and after 100 cycles plotted against scan rates. The capacitance currents were measured at 150 mV (vs. NHE).

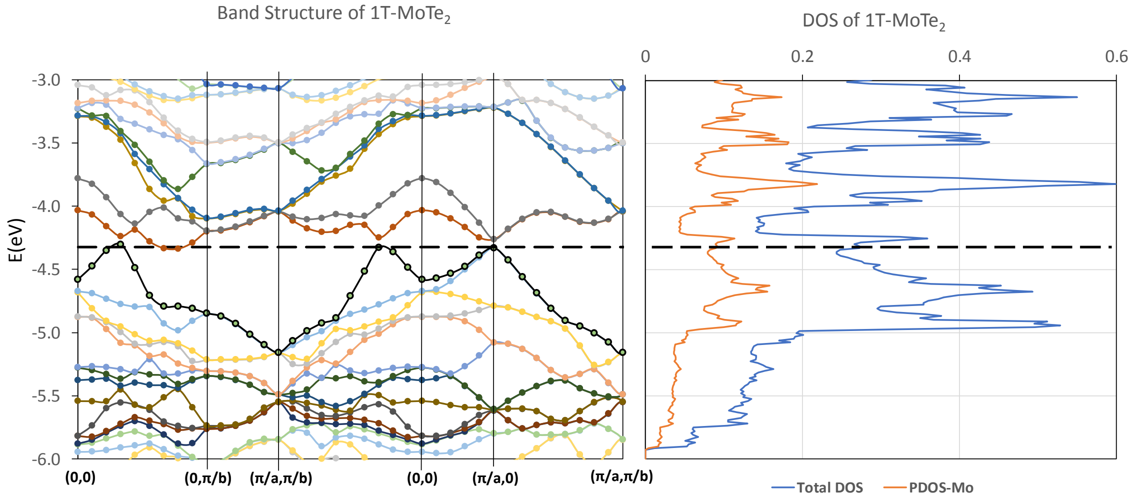

Supplementary Figure 20: Band structure and Density of States (DOS) of an optimised 2x2 unit cell of 1T'-$MoTe_2$.In orange is the projected density of states associated with the molybdenum atomic orbitals. The dashed black line shows the position of the Fermi level.

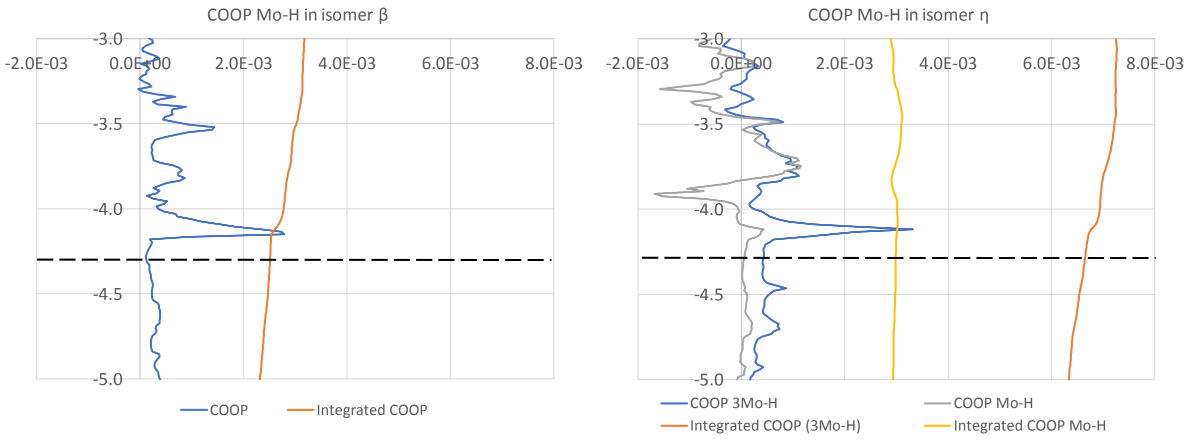

Supplementary Figure 21: Comparison of Crystal Orbital Overlap Populations (COOP) and respective integrated values for $^{2}_{\infty}$[β-MoTe$_2$H$_{0.125}$] and $^{2}_{\infty}$[η-MoTe$_2$H$_{0.125}$].The dashed line corresponds to the Fermi level.

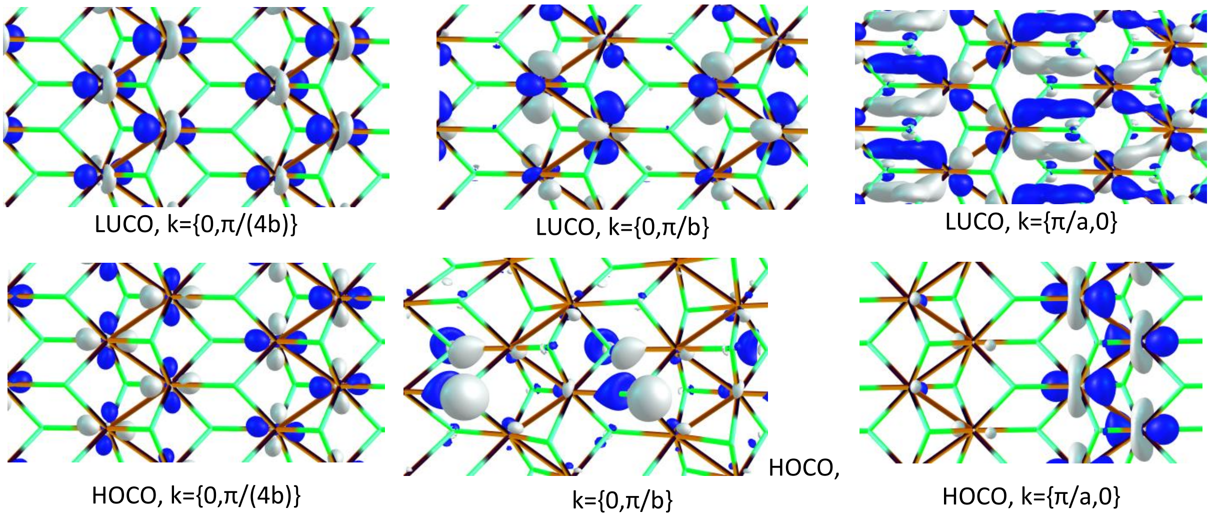

Supplementary Figure 22: Top view of selected crystal orbitals of the 2x2 unit cell of 1T'-$MoTe_2$ located at the crossing between the valence and conduction bands (Fermi level).

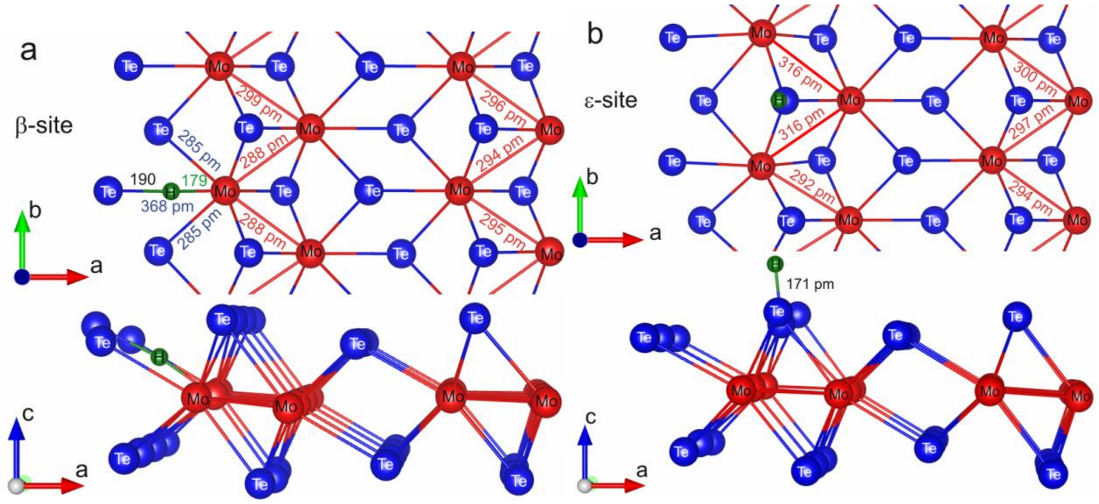

Supplementary Figure 23: Optimised structures and bond distances (pm) of the regio-isomers of $^{2}_{\infty}[\mathbf{MoTe_2H_{0.125}}]$ (2x2 unit cell) with hydrogen adsorbed at (a) β-site and (d) ε-site.

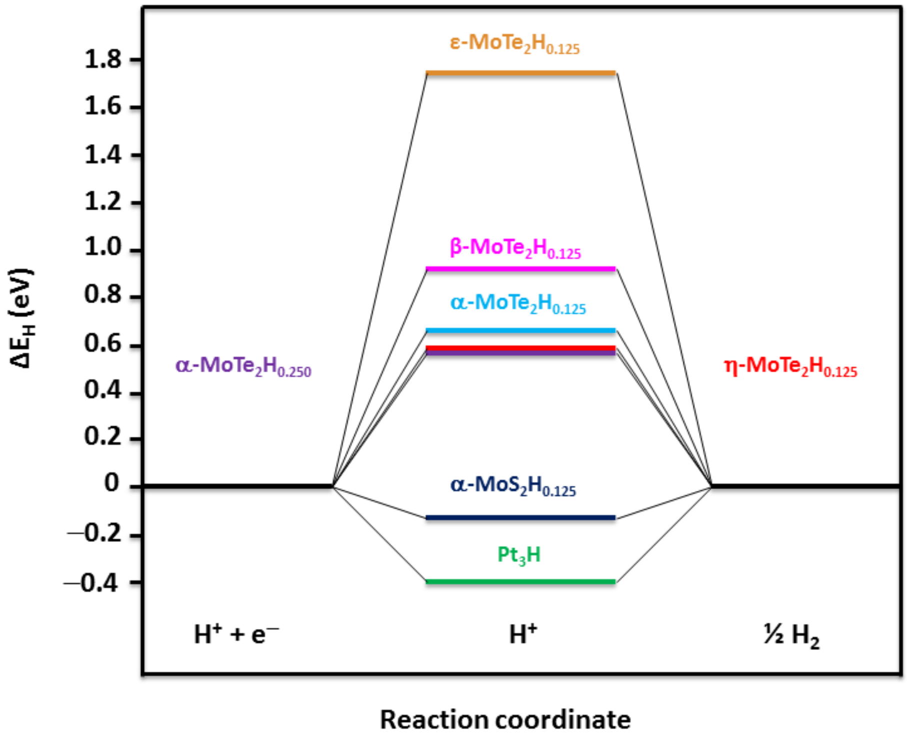

Supplementary Figure 24: Comparison of ΔE_H (eV) values at various H-bonding sites.

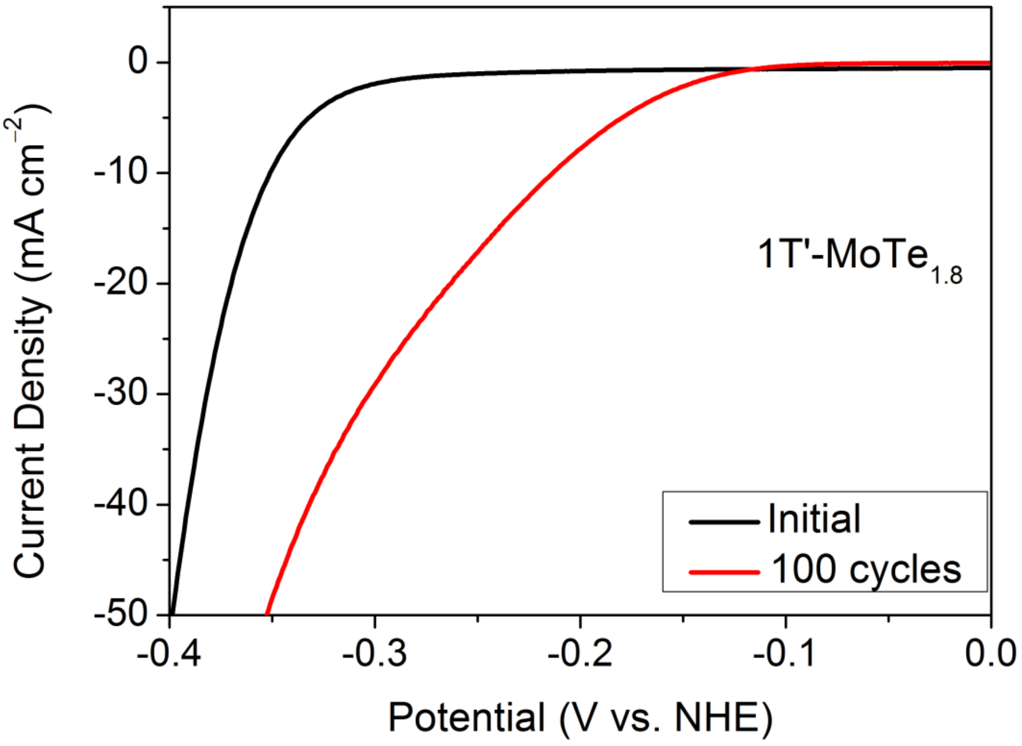

Supplementary Figure 25: Comparison of the current densities achieved by Te-deficient nanocrystalline 1T'-$MoTe_{1.8}$ before and after 100 cycles in 1 M H_2SO_4. Catalysts were prepared on a glassy carbon working electrode as described in the experimental section. Carbon felt and 3 M Ag/AgCl were used as the counter and reference electrodes, respectively.

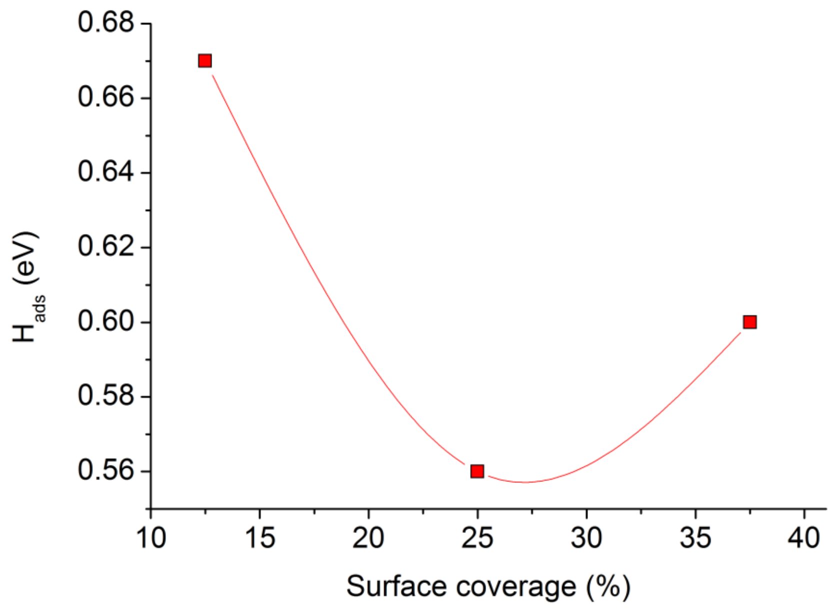

Supplementary Figure 26: The hydrogen adsorption energy on the α-site as a function of $MoTe_2$ surface coverage.

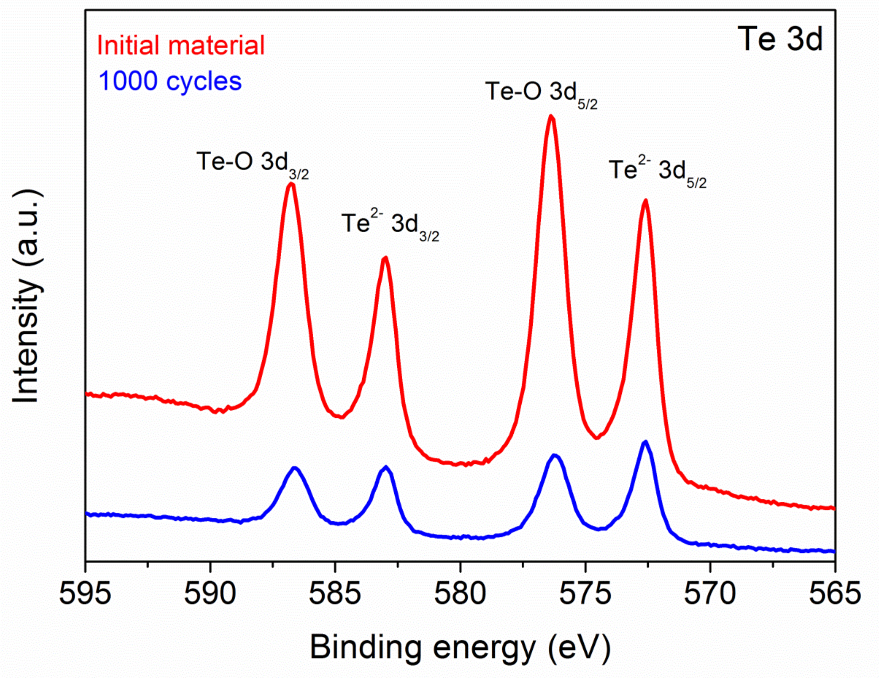

Supplementary Figure 27: High resolution XPS spectra of the Te 3d regions of nanocrystalline 1T'-$MoTe_2$ before and after 1000 cycles for studies carried in ambient conditions.

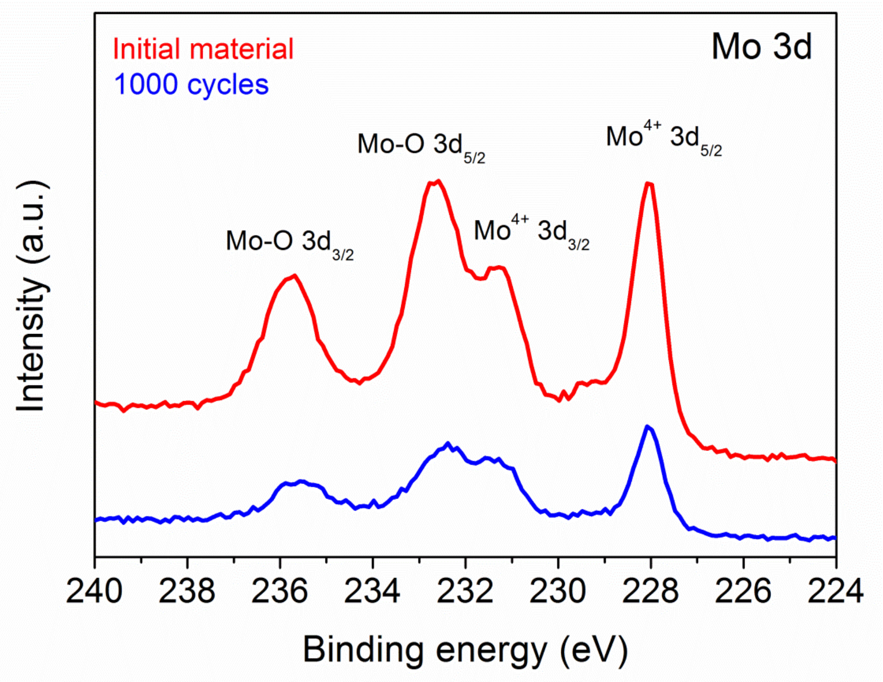

Supplementary Figure 28: High resolution XPS spectra of the Mo 3d regions of nanocrystalline 1T'-$MoTe_2$ before and after 1000 cycles for studies carried in ambient conditions.

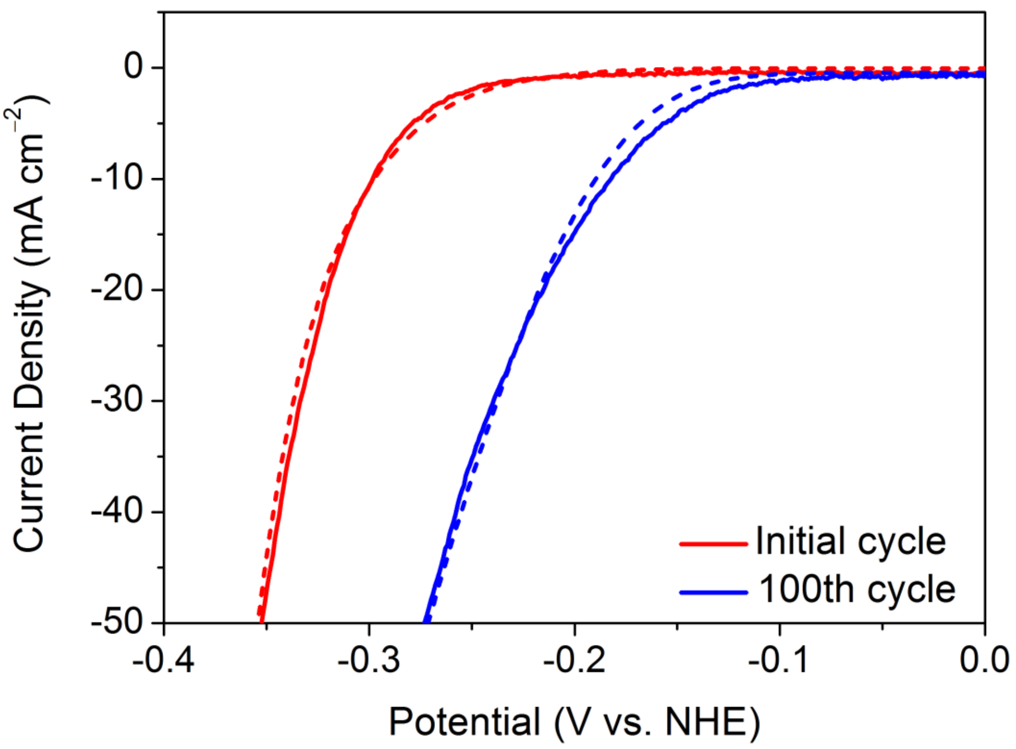

Supplementary Figure 29: Comparison of the current densities achieved by nanocrystalline 1T'-$MoTe_2$ before and after 100 cycles in 1 M H_2SO_4 under ambient conditions (solid lines) and when handled under inert atmosphere (dashed lines). Catalysts were prepared on a glassy carbon working electrode as described in the experimental section. Carbon felt and 3 M Ag/AgCl were used as the counter and reference electrodes, respectively.

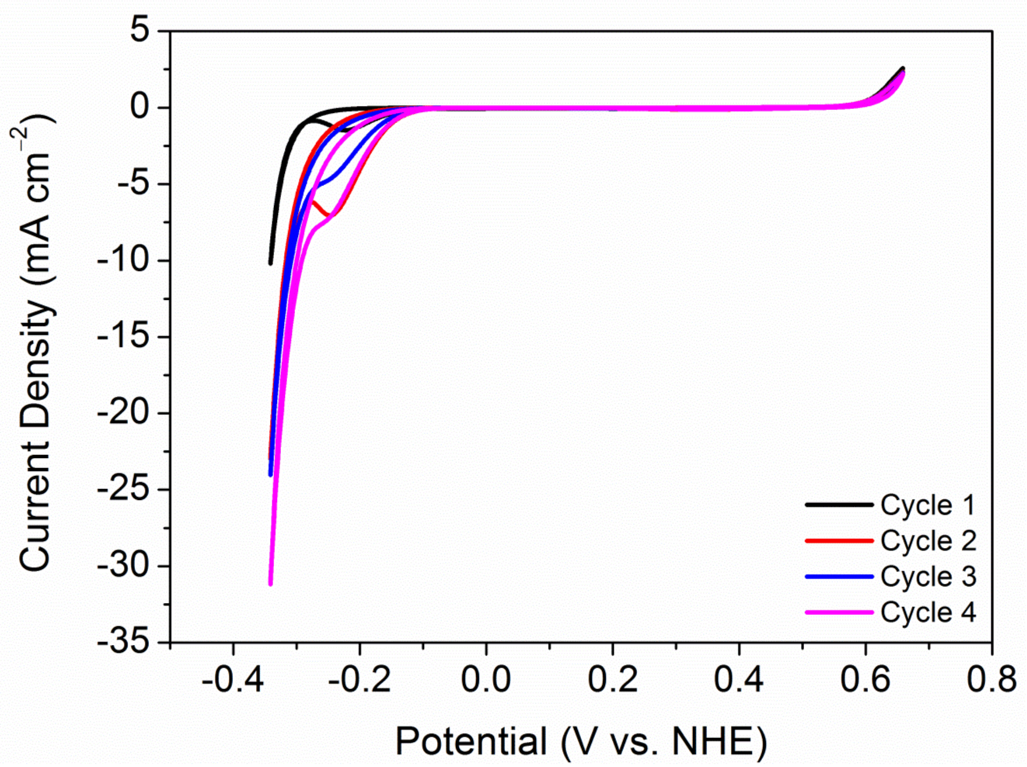

Supplementary Figure 30: Cyclic voltammogram of the edge site oxidation of nanocrystalline 1T'-$MoTe_2$ and subsequent catalytic enhancement indicating the edges have no role in the activation process. Measurements were performed in 1 M H_2SO_4 under a nitrogen atmosphere with a scan rate of 10 mV s^{-1}.

Supplementary Tables

Supplementary Table 1: Comparison of HER activity in state-of-the-art catalysts

Catalyst	V (vs. NHE) at *j* = 10 mA cm^{-2}	Tafel Slope (mV dec^{-1})	Reference
Double gyroid MoS_2	0.28 V	50	2
MoS_2 Nanoparticles	0.17 V	55-60	3
Exfoliated 1T-MoS_2 Nanosheets	0.19 V	43	4
MoS_2/RGO	0.16 V	41	5
Amorphous MoS_2	0.20 V	60	6
$[Mo_3S_{13}]^{2-}$ nanoclusters on carbon supports	0.18 – 0.22 V	38-57	7
Cu_7S_4@MoS_2 Hetero-nanoframes	0.13 V	48	8
Defect-rich MoS_2 nanosheets	0.2 V	50	9
T-WS_2 nanosheets	0.23	60	10
WS_2/RGO	0.26 V	58	11

Supplementary Table 2: Summary of the parameters used for estimations of TOF and surface coverage

	**Initial**	**After 100 cycles**
**Exchange current (*i_0*), Amperes**	7.33×10^{-9}	3.57×10^{-5}
**Total moles of H_2 per *s* (*n*), mol / *s***	3.80×10^{-14}	1.85×10^{-10}
**TOF, sites / *s***	2.53×10^{-5}	0.123
**Surface coverage, %**	$2.53 x 10^{-3}$	12.3

Supplementary Table 3: Summary of the raw data obtained by ICP-OES. ICP-OES was performed on the as prepared catalyst ink material before cycling, and after 100 cycles. Each measurement was repeated three times, with the compositions averaged over the three samples.

Sample	Before cycling	After 100 cycles
Crystalline $MoTe_2$	$MoTe_{2.023}$	$MoTe_{2.037}$
	$MoTe_{2.042}$	$MoTe_{2.033}$
	$MoTe_{2.052}$	$MoTe_{2.050}$
Nanocrystalline $MoTe_2$	$MoTe_{1.912}$	$MoTe_{1.979}$
	$MoTe_{1.993}$	$MoTe_{2.002}$
	$MoTe_{2.002}$	$MoTe_{1.966}$

Supplementary Table 4: DFT Optimised (rev-PBE-D3) structural parameters of the 2×2 unit cells with chemisorption energies. Experimental parameters for 1T'-$MoTe_2$ are listed in the first row in brackets.[1]

Structure	Unit cell parameters			ΔE_H(eV)	
	a (pm)	*b* (pm)	θ (°)	This work	Ref [1]
$^{2}_{\infty}[MoTe_2]$	129.9 (*126.6*)	674.9 (*693.8*)	90.0 (90)		
$^{2}_{\infty}[\alpha\text{-}MoTe_2H_{0.125}]$	129.71	684.0	89.9	+0.67	*+0.55*
$^{2}_{\infty}[\beta\text{-}MoTe_2H_{0.125}]$	131.59	673.8	90.0	+0.92	*+0.82*
$^{2}_{\infty}[\varepsilon\text{-}MoTe_2H_{0.125}]$	130.72	679.7	90.0	+1.73	*+1.43*
$^{2}_{\infty}[\eta\text{-}MoTe_2H_{0.125}]$	130.63	674.9	90.0	+0.58	

Supplementary Table 5: Hydrogen adsorption energy relationship to the logarithm of the exchange current.

Surface	ΔE_H (eV)	log (i_0/A.cm^{-2})	Tafel slope (mV dec^{-1})	Ref.
α-$^{\infty}_{2}[Mo_8Te_{16}H]$	+0.67	-6.99	68	This work
α-$^{\infty}_{2}[Mo_8S_{16}H]$	-0.13	-4.90	48	9
$^{\infty}_{2}[Pt_3H]$	-0.40	-3.34	30	12

Supplementary Discussion

Synthesis and characterisation of nanocrystalline 1T'-$MoTe_2$

Reaction between Mo and Te at temperatures of 400 °C allows for the formation of a black material with a diffraction pattern (Supplementary Fig. 1) similar to the one collected on a sample prepared by regrinding single crystals of the high temperature monoclinic 1T'-$MoTe_2$ phase and literature reports.[13,14,15] The pattern also matches well with the nanocrystalline $MoTe_2$ products synthesised recently *via* a solution based route.[16] It is apparent that the low synthetic temperature results in a more disordered material (referred to as nanocrystalline 1T'-$MoTe_2$ throughout the text), which is easily distinguishable from its high temperature variant (referred to as crystalline 1T'-$MoTe_2$) by substantially broader peaks. Although the solution-based route has previously been demonstrated as a viable strategy for synthesis of 1T'-$MoTe_2$ at 300°C,[16] the formation of this metallic phase by a solid state route instead of the more stable at low temperature semiconducting polymorph of $MoTe_2$ with the hexagonal structure is remarkable. Therefore, Raman spectroscopy was used to confirm that the coordination environment in the synthesised material was indeed consistent with the distorted {$MoTe_6$} octahedra, which are the expected building blocks for the monoclinic phase (Supplementary Fig. 2). For nanocrystalline 1T'-$MoTe_2$ the Raman peaks observed at 110, 127, 163 and 256 cm^{-1} correspond to the A_g- and B_g- modes and are in very close agreement with the peaks observed in the Raman spectrum recorded on a single crystal of crystalline monoclinic 1T'-$MoTe_2$ and literature data.[17,18] The wide range spectra also highlights the absence of MoO_2 and MoO_3, thus confirming that both materials are free from oxide impurities in the bulk.[19,20]

TEM investigations of the nanocrystalline 1T'-$MoTe_2$ product revealed the formation of agglomerates of fragmented crystallites (Supplementary Fig. 3a) with sizes in the range of ~10 nm as demonstrated by high-magnification TEM images (Supplementary Fig. 3b). Electron diffraction patterns collected from the crystallites confirm the validity of the

proposed model, *e.g. 1T'-MoTe$_2$* with the monoclinic distortion. Thus, the formation of 1T'-MoTe$_2$ polymorph is confirmed (Supplementary Fig. 3c-e) and is in good agreement with the data reported for nanocrystalline 1T'-MoTe$_2$ by Schaak's group.[16] Further twins with twin boundary (100) and respective splitting of planes along *a**-direction were found which are prototypical for the para-variant twin of 1T'-MoTe$_2$.[21] In comparison, the crystalline 1T'-MoTe$_2$ exhibited substantially larger crystallites evident as platelets with the sizes in the range of several micrometres (Supplementary Fig. 4a). Electron diffraction patterns were found in good agreement with expected monoclinic structure as well (Supplementary Fig. 4b-d).

The normalised XANES spectra (Supplementary Fig. 5) of both crystalline 1T'-MoTe$_2$ and nanocrystalline 1T'-MoTe$_2$ resemble the literature spectra of MoS$_2$, with the main edge at 2006 eV, consistent with Mo^{4+} oxidation state and a sharp feature at 2015 eV.[22] From the EXAFS data, there is a slight decrease in intensity of the oscillations of nanocrystalline 1T'-MoTe$_2$ compared with crystalline 1T'-MoTe$_2$, which could indicate a slight decrease in number of neighbours, as observed from the feature at ~2.5 Å in the non-phase corrected plot of the k^2 weighted Fourier Transform of the EXAFS data (Supplementary Fig. 5), The EXAFS data can be fitted using two Mo-Te paths at 2.7 and 2.8 Å and a Mo-Mo path at 3.46Å. The magnitude and imaginary components of the k^2-weighted Fourier transform data and fits of crystalline and nanocrystalline 1T'-MoTe$_2$ are shown in Supplementary Fig. 6, with the imaginary components of each scattering path used. Despite the subtle differences observed in the EXAFS data, no statistically significant difference can be observed between the two samples, the Mo-Te coordination numbers and distances are within error.

Electrochemical performance of nanocrystalline 1T'-MoTe$_2$ before activation

The confirmation of the distorted [MoTe$_6$] octahedral environment in nanocrystalline 1T'-MoTe$_2$ suggests that it could demonstrate a good electrochemical performance, by drawing the analogy from the previous report on electrocatalytic activity in crystalline 1T'-MoTe$_2$.[23] Therefore, the activity of the nanocrystalline 1T'-MoTe$_2$ material for the electrochemical

hydrogen evolution reaction was assessed in 1 M H_2SO_4 and the sample revealed a good performance from the outset (red line in Supplementary Fig. 10). Moving on from the previously reported crystalline 1T'-$MoTe_2$, the nanocrystalline phase displays similar overpotential of 320 mV at $j = -10$ mA cm^{-2}. The similarity of the overpotential is important since it confirms that the activation process does not stem from the possible excess of edge sites. If the substantially improved overpotential observed after activation were due to additional edge sites then we should observe it on the initial cycle. Given that an identical catalyst loading per geometrical surface area was used on both electrodes, the marginal difference in activity between crystalline and nanocrystalline 1T'-$MoTe_2$ must stem from the differences in surface morphology of the two materials. The synthetic procedure of nanocrystalline 1T'-$MoTe_2$, which provides a more disordered arrangement of the layers, would result in a greater number of active sites accessible for a catalytic reaction. This is in line with the contrasting particle morphologies as can be seen from the TEM (Supplementary Fig. 3) and also SEM images (Supplementary Fig. 7) which clearly illustrate large single crystals of crystalline 1T'-$MoTe_2$ whilst the nanocrystalline phase exhibits a more disordered material with a larger surface area. Further, gas chromatography confirmed the reduction corresponded to the evolution of hydrogen with a Faradaic Efficiency of 96 ± 6% (Supplementary Fig. 12).

Exchange current density, Turnover Frequencies and Surface Coverage:

The exchange current densities (i_0) for 1T'-$MoTe_2$ were estimated by extrapolation the Tafel slopes on 0 mV. In the case of the non-activated, initial material the observed value was 1.03×10^{-4} mA cm^{-2}. The activated material showed a substantially higher value of 5.03×10^{-1} mA cm^{-2}. It should be mentioned that the current densities were calculated per geometrical area of the electrode which was 0.071 cm^2.

Catalytic activity can be quantified in terms of the turnover frequency (TOF). TOFs represent the number of reactant molecules converted on catalytic sites into the desired product over

time. Under assumption that each catalytic site would produce a single H_2 molecule we could calculate the TOF using Supplementary Equation 1:

$$TOF = \frac{n\,(mol\,s^{-1})\times N_A(molecules\,mol^{-1})\times S(cm^2)}{A\,(cm^2)} \quad (1)$$

where i_0 is the exchange current, S= 2.22×10^{-15} cm^2 is the surface area of the unit cell of the 1T'-$MoTe_2$ in cm^2; A = 2 cm^2 is the total surface area of the catalyst available for HER; n is number of moles of H_2 produced per second calculated using the Faraday equation at a given i_0; N_A is Avogadro constant. The results for initial and activated (after 100 cycles) materials are summarised in Supplementary Table 2. To calculate A we used the BET surface area of 1.8 m^2/g or 18 cm^2/mg obtained by N_2 adsorption measurements on nanocrystalline 1T'-$MoTe_2$. At the catalyst loading of 0.3 mg the total surface area of the catalyst corresponds to 6 cm^2. A further assumption was made that only 1/3 of the surface would be available for electrocatalytic reaction due to overlaps between platelets within the material. As such, A is an estimate rather than an exact value.

The TOF is measured in molecules per second and despite being expressed as a fractional number it implies that only a certain number of sites on the surface of the $MoTe_2$ is available for catalytic reaction. Therefore, it is better to express the TOF through the surface coverage which would represent the integer number of sites available. For example, the surface coverage of 12.3 % for product after 100 cycles would correspond to roughly one of every eight catalytic sites available for catalytic reaction at a given time.

Computational studies

The bonding energy of a surface to a hydrogen atom can be directly correlated to the activation energy of the reduction step.[12] From an experimental standpoint this is expressed by the electrochemical overpotential. Therefore, it is useful to compute the binding energies to infer and predict the optimum catalyst and additionally obtain an atomistic insight into the catalytic process. A 2x2 supercell of a monolayer slab was adapted as working models of 1T'-$MoTe_2$ (Fig. 4a). The optimised unit cell parameters (a = 649.2 pm, b = 337.5 pm) for the

monolayer surface are comparable with experimentally reported (a = 633 pm, b = 346.9 pm) and calculated values (a = 652 pm, b = 352 pm).[13] The slight expansion of the a-parameter is likely due to solvation effects. The values for the binding energies (Supplementary Table 4) should be examined with caution since they result from some approxiamtions on the surface binding site. For instance, charge effects caused by the electrode potential are absent.[24] However, they are sufficiently relevant to establish the stereochemistry of hydrogen adsorption.

The surface chemistry of 1T'-$MoTe_2$ is dictated by the frontier orbitals especially the ones at the crossing point between the valence and conduction regions. The band structure plot (

Supplementary Figure 20) indicates that there are three such points along the irreducible wedge of the Brillouin zone: at around k={0,π/(4b)}, {π/(4a),π/(4b)} and {π/a,0}. The {π/a,π/b} to {0,0} path is an approximate mirror image of the {0,0} to {0,π/b} segment, a sign of nodal plane degeneracy and approximately identical atomic orbital composition along both paths.

The Highest Occupied Crystal Orbital (HOCO) at k={0,π/(4b)} is displayed in Supplementary Fig. 21 and it is a zig-zag linear combination of in-phase 4d-orbitals of σ symmetry. The Lowest Unoccupied counterpart (LUCO) is a linear combination of 4d-orbitals of π symmetry perpendicular to the surface plane.

At k={π/a,0} another set of 4d orbitals are present, the HOCO being similar in nature to the one at k={0,π/(4b)} except they are slightly tilted above and below the surface plane in a zig-zag manner which is the result of a slight admixing with the π symmetry set of AOs. The LUCO is altogether different in composition and is a linear combination involving metal to ligand π-backdonation. This virtual crystal orbital belongs to a band which travels across the Fermi level into an electronically occupied region and becomes the HOCO-2 at k={π/a,π/b}. The tellurium lone pairs that are perpendicular to the basal plane have their highest energy at k={0,π/b}, are local (*i.e.* do not cross into the conduction region) and are involved in

forming the Mo-Te bonds.

The three H–Mo bond distances in $^{2}_{\infty}[\eta\text{-MoTe}_2\text{H}_{0.125}]$ are 185 pm and are only slightly longer than the 179 pm seen in β, but bond lengths do not necessarily correlate with bond strengths. A better quantitative measure is the integration of the crystal orbital overlap population[25] between atomic fragments (i,j) over all the range of energies up to the Fermi level, as shown in Supplementary Equation 2:

$$Int.COOP = \int_{-\infty}^{n} 2 \sum_{i,j} c_i(k) c_j(k) S_{i,j}(k)\, dE \qquad (2)$$

This value is a bond order metric between any given set of fragments as it accounts for the sum of the bonding and antibonding interactions between the fragment orbitals. For the metal hydride adsorbates this quantity was calculated and is presented in Supplementary Supplementary Figure 21. Two sets of values of COOP were calculated with the $^{2}_{\infty}[\eta\text{-MoTe}_2\text{H}_{0.125}]$ structure: one between the hydride and one nearest neighbour Mo site and between the hydride and the sum of three neighbouring Mo sites. It may be seen that the integrated COOP value is 3.0×10^{-3} for the former and 6.6×10^{-3} for the latter. For the $^{2}_{\infty}[\beta\text{-MoTe}_2\text{H}_{0.125}]$ isomer the corresponding integral of COOP(Mo-H) value is 2.5×10^{-3}. This is a clear indication that the three bonds are largely equivalent in strength.

Additional Experimental Details of Computational Studies

More accurate single point energy evaluations of the obtained stationary points were calculated using a denser 5×9 k point grid on the optimised unit cells (Scheme 1).

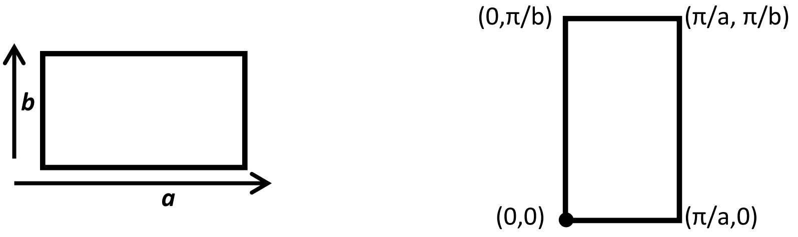

Scheme 1: Sketch of the real space unit cell and the corresponding main symmetry points in the Brillouin zone for an approximately rectangular lattice where a>b. The reciprocal lattice was sampled with 5 k points along a^{-1} and 9 k points along b^{-1}.

Crystal orbital overlap populations were calculated using a grid of 2000 points in the range of –0.7 to +0.4 a.u. below and above the Fermi levels respectively.

Electrochemical activation of crystalline 1T'-$MoTe_2$

Upon cycling crystalline 1T'-$MoTe_2$ under reducing potentials, a similar activation is observed, albeit to a lesser extent most likely due to the bulk plate-like microcrystals which may hinder access of protons to the active sites (Supplementary Fig. 11). Therefore, it is evident that the morphology of 1T'-$MoTe_2$ plays a key role in the activation process. Furthermore, EIS indicates higher conductivity for the nanocrystalline 1T'-$MoTe_2$ compared with the crystalline phase (Supplementary Fig. 14). This is in line with optical spectroscopy absorbance values, which are proportional to the optical conductivity and thus indicate a conductivity increase when going from the crystalline to the nanocrystalline phase (Supplementary Fig. 8). This means that electron transport is different between the two phases, likely due to the shorter interlayer pathways associated with the disordered nature of the nanocrystalline 1T'-$MoTe_2$ material.

Supplementary References

1. Seok, J. *et al.* Active hydrogen evolution through lattice distortion in metallic $MoTe_2$. *2D Mater.* **4,** 25061 (2017).

2. Kibsgaard, J. *et al.* Engineering the surface structure of MoS_2 to preferentially expose active edge sites for electrocatalysis. *Nat Mater.* **11,** 963–969 (2012).

3. Jaramillo, T. F. *et al.* Identification of Active Edge Sites for Electrochemical H2 Evolution from MoS_2 Nanocatalysts. *Science (80-.).* **317,** 100–102 (2007).

4. Lukowski, M. A. *et al.* Enhanced hydrogen evolution catalysis from chemically exfoliated metallic MoS_2 nanosheets. *J. Am. Chem. Soc.* **135**, 10274-10277 (2013).

5. Li, Y. *et al.* MoS_2 nanoparticles grown on graphene: An advanced catalyst for the hydrogen evolution reaction. *J. Am. Chem. Soc.* **133**, 7296-7299 (2011).

6. Benck, J. D. *et al.* Amorphous Molybdenum Sulfide Catalysts for Electrochemical Hydrogen Production: Insights into the Origin of their Catalytic Activity. *ACS Catal.* **2**, 1916-1923 (2012).

7. Kibsgaard, J. *et al.* Building an appropriate active-site motif into a hydrogen-evolution catalyst with thiomolybdate $[Mo_3S_{13}]^{2-}$ clusters. *Nat. Chem.* **6,** 248–253 (2014).

8. Xu, J. *et al.* Ultrasmall Cu_7S_4@MoS_2 Hetero-Nanoframes with Abundant Active Edge Sites for Ultrahigh-Performance Hydrogen Evolution. *Angew. Chem. Int. Ed.* **55**, 6502-6505 (2016).

9. Yin, Y. *et al.* Contributions of Phase, Sulfur Vacancies, and Edges to the Hydrogen Evolution Reaction Catalytic Activity of Porous Molybdenum Disulfide Nanosheets. *J. Am. Chem. Soc.* **138**(25), 7965-7972.

10. Voiry, D. *et al.* Enhanced catalytic activity in strained chemically exfoliated WS_2 nanosheets for hydrogen evolution. *Nat. Mater.* **12**, 850-855 (2013).

11. Yang, J. *et al.* Two-Dimensional Hybrid Nanosheets of Tungsten Disulfide and Reduced Graphene Oxide as Catalysts for Enhanced Hydrogen Evolution. *Angew. Chem. Int. Ed.* **52**, 13751-13754 (2013).

12. Nørskov, J. K. *et al.* Trends in the Exchange Current for Hydrogen Evolution. *J. Electrochem. Soc.* **152,** J23–J26 (2005).

13. Brown, B. E. The Crystal Structures of WTe_2 and High-Temperature $MoTe_2$. *Acta Cryst.* **20,** 268 (1966).

14. Keum, D. H. *et al.* Bandgap opening in few-layered monoclinic $MoTe_2$. *Nat. Phys.* **11,** 482–486 (2015).

15. Vellinga, M. B., de Jonge, R. & Haas, C. Semiconductor to Metal Transition in $MoTe_2$. *J. Solid State Chem.* **2,** 299–302 (1970).

16. Sun, Y. *et al.* Low-Temperature Solution Synthesis of Few-Layer 1T'-$MoTe_2$ Nanostructures Exhibiting Lattice Compression. *Angew. Chem. Int. Ed.* **55,** 2830–2834 (2016).

17. Jana, M. K. *et al.* Structure and Electron-Transport Properties of Anion-Deficient $MoTe_2$: A Combined Experimental and Theoretical Study. *Z. Anorg. Allg. Chem.* **642**

(23), 1386-1396 (2016).

18. Zhou, L. *et al.* Sensitive Phonon-Based Probe for Structure Identification of 1T′ $MoTe_2$. *J. Am. Chem. Soc.* **139,** 8396–8399 (2017).

19. Pine, A. S. & Dresselhaus, G. Raman Spectra and Lattice Dynamics of Tellurium. *Phys. Rev. B* **4,** 356–371 (1971).

20. Zhou, L. *et al.* Synthesis of High-Quality Large-Area Homogenous 1T′ $MoTe_2$ from Chemical Vapor Deposition. *Adv. Mater.* **28,** 9526–9531 (2016).

21. Manolikas, C. & Spyridelis, J. Electron microscopy and electron diffraction study of the domain structure and the transition state in $CuInSe_2$. *Mater. Res. Bull.* **16,** 501–504 (1981).

22. Cesano, F. *et al.* Model oxide supported MoS_2 HDS catalysts: Structure and surface properties. *Catal. Sci. Technol.* **1,** 123–136 (2011).

23. McGlynn, J. C. *et al.* Molybdenum Ditelluride Rendered into an Efficient and Stable Electrocatalyst for the Hydrogen Evolution Reaction by Polymorphic Control. *Energy Technol.* **6,** 345–350 (2018).

24. Donghoon, K., Shi, J. & Liu, Y. Substantial Impact of Charge on Electrochemical Reactions of Two-Dimensional Materials. *J. Am. Chem. Soc.* **140**, 9127-9131 (2018).

25. Hoffmann, R. *Solids and Surfaces : A Chemist’s View of Bonding in Extended Structures*. (VCH Publishers, Inc, 1988).
